# Phosphorylation of the Amyloid-Beta Peptide Inhibits Zinc-Dependent Aggregation, Prevents Na,K-ATPase Inhibition, and Reduces Cerebral Plaque Deposition

**DOI:** 10.3389/fnmol.2018.00302

**Published:** 2018-08-29

**Authors:** Evgeny P. Barykin, Irina Y. Petrushanko, Sergey A. Kozin, Georgy B. Telegin, Alexander S. Chernov, Olga D. Lopina, Sergey P. Radko, Vladimir A. Mitkevich, Alexander A. Makarov

**Affiliations:** ^1^Engelhardt Institute of Molecular Biology, Russian Academy of Sciences, Moscow, Russia; ^2^Pushchino Branch of Shemyakin-Ovchinnikov Institute of Bioorganic Chemistry, Russian Academy of Sciences, Pushchino, Russia; ^3^Faculty of Biology, Lomonosov Moscow State University, Moscow, Russia; ^4^Institute of Biomedical Chemistry, Russian Academy of Sciences, Moscow, Russia

**Keywords:** Alzheimer’s disease, β-amyloid, Na^+^, K^+^-ATPase, zinc, protein phosphorylation, amyloid plaques, aggregation, metal-binding domain

## Abstract

The triggers of late-onset sporadic Alzheimer’s disease (AD) are still poorly understood. Impairment of protein phosphorylation with age is well-known; however, the role of the phosphorylation in β-amyloid peptide (Aβ) is not studied sufficiently. Zinc-induced oligomerization of Aβ represents a potential seeding mechanism for the formation of neurotoxic Aβ oligomers and aggregates. Phosphorylation of Aβ by Ser8 (pS8-Aβ), localized inside the zinc-binding domain of the peptide, may significantly alter its zinc-induced oligomerization. Indeed, using dynamic light scattering, we have shown that phosphorylation by Ser8 dramatically reduces zinc-induced aggregation of Aβ, and moreover pS8-Aβ suppresses zinc-driven aggregation of non-modified Aβ in an equimolar mixture. We have further analyzed the effect of pS8-Aβ on the progression of cerebral amyloidosis with serial retro-orbital injections of the peptide in APPSwe/PSEN1dE9 murine model of AD, followed by histological analysis of amyloid burden in hippocampus. Unlike the non-modified Aβ that has no influence on the amyloidosis progression in murine models of AD, pS8-Aβ injections reduced the number of amyloid plaques in the hippocampus of mice by one-third. Recently shown inhibition of Na^+^,K^+^-ATPase activity by Aβ, which is thought to be a major contributor to neuronal dysfunction in AD, is completely reversed by phosphorylation of the peptide. Thus, several AD-associated pathogenic properties of Aβ are neutralized by its phosphorylation.

## Introduction

The amyloid-beta peptide (Aβ) is a normal subnanomolar component of biological fluids (Masters and Selkoe, 2012); however, its deposition in the form of amyloid plaques is one of the hallmarks of AD (Selkoe and Hardy, 2016). Amyloid plaques are associated with neuronal loss and cognitive impairment (Musiek and Holtzman, 2015), and they enhance the tau pathology (He et al., 2017). The trigger of the pathologic Aβ aggregation in AD is unknown (Musiek and Holtzman, 2015); however, in animal models of AD, the conversion of a monomeric Aβ into fibrillar aggregates, through neurotoxic oligomers, is triggered by chemically and structurally modified Aβ species (Meyer-Luehmann et al., 2006; Prusiner, 2012; Jucker and Walker, 2013). Amyloid plaques are abnormally rich in Fe, Cu, and Zn ions (Cummings, 2004), and data from animal models suggest that the formation of amyloid plaques is zinc dependent (Friedlich et al., 2004; Frederickson et al., 2005; DeGrado et al., 2016; James et al., 2017). It has been assumed that zinc ions promote Aβ aggregation via a population shift of polymorphic states (Miller et al., 2010). Zinc-induced aggregation of Aβ is governed by its metal-binding domain (Aβ_16_), which comprises the N-terminal region (residues 1–16) of Aβ (Istrate et al., 2016). It has recently been shown that the metal-binding domain of Aβ containing isomerized Asp7 (isoD7-Aβ_16_) is more prone to zinc-induced oligomerization (Istrate et al., 2016), suggesting a role for Asp7 isomerization in the initiation of the pathological aggregation process. Indeed, a full-length isoD7-Aβ_42_ peptide is more neurotoxic than the unmodified peptide (Mitkevich et al., 2013) and is able to trigger cerebral amyloidosis *in vivo* (Kozin et al., 2013). Thus, the ability of Aβ to form zinc-induced aggregates may correlate with its amyloidogenic and neurotoxic properties.

Recently, it has been shown that Aβ undergoes phosphorylation at Ser8 both *in vitro* and *in vivo* (Kumar et al., 2011, 2013). In the absence of zinc ions, phosphorylated Aβ (pS8-Aβ) was found to be a fast-aggregating peptide species, producing stable fibrillar aggregates and neurotoxic oligomers *in vitro* (Rezaei-Ghaleh et al., 2016; Jamasbi et al., 2017). In the presence of Zn^2+^, the metal-binding domain of pS8-Aβ (pS8-Aβ_16_) forms stable dimers (Kulikova et al., 2014); however, in contrast to the zinc-mediated behavior of similar domains in several other Aβ isoforms (including native Aβ), the zinc-bound dimers of pS8-Aβ_16_ do not give rise to larger oligomers and aggregates (Istrate et al., 2016). Moreover, the zinc-driven heterodimers formed between pS8-Aβ_16_ and isoD7-Aβ_16_ or Aβ_16_ also cannot oligomerize. We hypothesized that pS8-Aβ_42_ does not aggregate in the presence of zinc ions and that pS8-Aβ_42_ can prevent the aggregation of native Aβ through the formation of non-propagating heterodimers between pS8-Aβ_42_ and Aβ (Mezentsev et al., 2016). By contrast, phosphorylation at Ser8 may change the interaction of Aβ with other proteins, such as Na^+^,K^+^-ATPase. Previously, it was shown that Na^+^,K^+^-ATPase activity was inhibited in post-mortem tissues of AD patients and in amyloid-containing hippocampi of transgenic mice (but not in the amyloid-free cerebellum) (Dickey et al., 2005; Kreutz et al., 2013; Zhang et al., 2013). The latest studies demonstrated that Aβ_42_ in form of monomers or oligomers directly binds to Na^+^,K^+^-ATPase, which results in the inhibition of the enzyme as well as the triggering of intracellular signaling cascades (Ohnishi et al., 2015; Petrushanko et al., 2016). Therefore, phosphorylation at Ser8 might neutralize some pathogenic properties of Aβ. Furthermore, the concentration of pS8-Aβ is very likely to change with age since brain aging is associated with changes in the activity of kinases and phosphatases in nerve tissue (Jin and Saitoh, 1995; Norris et al., 1998). An age-related shift in the neuronal protein phosphorylation has recently been shown in *Drosophila* models (Thomas and Haberman, 2016). Thus, phosphorylated Aβ species could be significant for the development of late-onset AD. However, there are no published data on the zinc-dependent oligomerization and related properties of a full-length pS8-Aβ peptide. In the present study, we investigated the role of pS8-Aβ_42_ as a potential quencher of zinc-induced oligomerization of endogenous Aβ species and of pathological effects associated with this process, such as the inhibition of Na^+^,K^+^-ATPase and induction of cerebral amyloidosis, in AD model mice.

## Materials and Methods

### Host Mice

B6C3-Tg(APPswe,PSEN1dE9)85Dbo mice were used at the age of 2–8 months (weight of 24–26 g). Mice were housed in the Pushchino Animal Breeding Facility (branch of the Shemyakin and Ovchinnikov Institute of Bioorganic Chemistry, Russian Academy of Sciences), under specific pathogen-free conditions. Housing and use of laboratory animals were approved by the commission IACUC, protocol No. 479/15 from 16.03.15. All animal manipulations were performed according to recommendations of the Guide for the Care and Use of Laboratory Animals (NRC 2011), the European Convention for the Protection of Vertebrate Animals Used for Experimental and Other Scientific Purposes, Council of Europe (ETS 123). The experimental procedures were approved by the local Animal Care and Use Committee (Reg. No. 126/15 from March 31, 2015). The study was supported by the Federal Agency for Scientific Organizations Program for Support of the Bioresource Collections.

### Reagents

All chemicals and solvents used throughout this study were of HPLC-grade or better and were obtained from Sigma-Aldrich (St. Louis, MO, United States) unless otherwise stated. Synthetic peptides (purity [98%] checked by RP-HPLC) Aβ_42_ ([H2N]-DAEFRHDSGYEVHHQKLVFFAEDVGSNKGAIIGLMVGGVVIA-[COOH]) and pS8-Aβ_42_ ([H2N]-DAEFRHD[pS]GYEVHHQKLVFFAEDVGSNKGAIIGLMVGGVVIA-[COOH]) were purchased from Biopeptide (San Diego, CA, United States). The amino acid sequence of the peptide was confirmed with an ultra-high resolution Fourier transform ion cyclotron resonance mass-spectrometer 7T Apex Qe BRUKER (Bruker Daltonics, Bellerica, MA, United States) utilizing a *de novo* sequencing approach based on a CID fragmentation technique as described earlier (Indeykina et al., 2011).

### Aβ Peptides Preparations

#### Synthetic pS8-Aβ_42_ Preparations for Injections

Two thousand micrograms of peptide pS8-Aβ_42_ were dissolved in 2000 μL of sterile physiological saline (PS), then the prepared solution was filtered through a 0.22-μm filter (Millex-GV, Millipore), aliquoted to 125 μL and frozen. For injection, one aliquot was thawed, sterile PS was added to obtain 1500 μL of solution with a peptide concentration of 0.08333 μg/μL (“administration solution”). Then, 150 μL of “administration solution” were withdrawn and 125 μL of this solution were injected into one animal. Thus, with a single injection, 10 μg of the peptide were ingested.

The aggregation state of the synthetic peptide pS8-Aβ_42_ in the samples used for the injections, characterized by us using a standard test based on thioflavin T (Ban et al., 2003), did not change during the time of storage (1–8 months), and did not differ from that of the freshly prepared solutions of the corresponding peptides.

#### Synthetic Aβ_42_ and pS8-Aβ_42_ Preparations for Na,K-ATPase Studies

To prepare working solutions of Aβ_42_ and pS8-Aβ_42_ peptides, cold hexafluoroisopropanol (“Fluka”) was added to dry Aβ to a concentration of 1 mM and incubated for 60 min at room temperature. The resulting solution was then placed in ice for 10 min and transferred to non-siliconized microcentrifuge tubes (0.56 mg peptide per tube). The peptides in the tubes were vacuum-dried with Eppendorf Concentrator 5301. The resulting dry peptides were stored at −80°C. A fresh 2.5 mM Aβ solution was prepared by adding 20 μl of 100% anhydrous dimethyl sulfoxide (“Sigma”) to 0.56 mg of the peptide, followed by incubation for 1 h at room temperature.

#### Synthetic Aβ_42_ and pS8-Aβ_42_ Preparations for DLS Measurements

To measure the average diameter of Aβ aggregates, synthetic peptides Aβ_42_ and pS8-Aβ_42_ were treated with hexafluoroisopropanol, dried, and dissolved in 10 mM NaOH at concentration of 0.5 mM. Peptides were brought to required concentrations in 10 mM HEPES (pH 7.4) supplemented with 150 mM NaCl, using appropriate buffers.

### Dynamic Light Scattering

Dynamic light scattering measurements were carried out using a Zetasizer Nano ZS apparatus (Malvern Instruments, Ltd., United Kingdom) at 25°C in accordance with the manufacturer instruction. The instrument is equipped with a He-Ne laser source (λ = 632.8 nm) and operates in the back-scatting mode, measuring particle size in the range between 0.6 nm and 10 μm. The particle size distribution is estimated with a spherical approximation of particles, employing a CONTIN data analysis utility available as a part of the instrument software, and used to calculate the average particle diameter (*D*). The aggregation of Aβ_42_ and pS8-Aβ_42_ peptides was triggered by diluting peptide solutions with a ZnCl_2_-containing buffer so as to provide a twofold molar excess of zinc ions over peptides.

### Turbidimetry

A peptide/zinc mixture was placed in a BRAND UV microcuvette (BRAND GMBH, Germany) and its turbidity was monitored in time (at room temperature) as optical density at 405 nm, using an Agilent 8453E spectrophotometer (Agilent Technologies, United States). Turbidity measurements were started in 0.5 min after triggering the zinc-induced Aβ aggregation as described for the DLS experiments. The initial (zero time) points were measured in the absence of zinc ions, using 25-μM Aβ solutions.

### Intravenous Injections

Retro-orbital injections of the venous sinus in mice were performed according to Yardeni et al. (2011). Mice received one retro-orbital injection with 1-month intervals between injections. The contents of injections for each group of mice are described in **Table 1**. The mice were assigned to the various groups randomly.

**Table 1:** Suppression of congophilic amyloid plaques formation in the brain of B6C3-Tg(APPswe, PSEN1dE9)85Dbo/J transgenic mice.

	Transgenic mice		Injection		Brain sections	Number of amyloid plaques per section	Statistical significance
					
Group name	Number of animals (male + female)	Age at first injection (months)	Age at sacrifice (months)	Administered compound/total number of injections	Total number	In hippocampus	Versus PS
PS	4 (2+2)	2	8	PS (125 μl)	40	28.7 ± 4.6	–
pS8-Aβ_42_	5 (2+3)	2	8	Synthetic pS8- Aβ_42_ peptide (10 μg in 125 μl of PS)	50	18.3 ± 0.94	*P* < 0.05

### Histology and Immunohistochemistry

Euthanasia procedure was applied to 8-month-old mice. Mouse euthanasia was carried out by CO_2_ according to the IACUC-approved protocol with the use of automated CO_2_-box Bioscape (Germany). Mice were transcardially perfused with 50 mL of PBS, followed by 50 mL of 4% paraformaldehyde (PFA). Mouse brains were fixed in 10% formalin. Process for paraffin embedding was scheduled as follow: 75% ethanol overnight, 96% ethanol 5 min, 96% ethanol 10 min, 100% ethanol 10 min (two changes), ethanol–chloroform (1:1) 30 min, chloroform overnight. Paraffin embedding was performed at 60°C for 3 h (three changes). The embedding of tissues into paraffin blocks was done using Leica EG1160 device. Serial brain sections (8 um) were cut using microtome Leica RM2265 mounted onto slides. For deparaffinization, hydration and staining of the sections the following steps were done: slides were consistently put in xylene three changes (10 min each), 96% ethanol (5 min), 90% ethanol (2 min), 75% ethanol (2 min), H_2_O three changes (5 min each), Congo Red solution (5 min), potassium alkali solution, and water. The Immu-Mount medium (Thermo Scientific) was used for mounting.

Immunostaining was carried out as described elsewhere (Kozin et al., 2013). Briefly, sections were deparaffinized and after antigen retrieval by microwaving in citrate buffer washed in PBS and blocked with 10% goat serum in 0.04% Tween20 in PBS (T-PBS). Sections were incubated with primary antibody for 2 h at room temperature, washed thrice in T-PBS, incubated with secondary antibody for 1 h at room temperature followed by washing in T-PBS. The mouse anti-human Aβ monoclonal antibodies 6E10 (Covance, Dallas, TX, United States) diluted in the block solution 1:1000 were used as the primary antibodies, Alexa Fluor 488 goat anti-mouse antibodies (Invitrogen, Grand Island, NY, United States) were used as the secondary antibodies for immunofluorescence staining (dilution 1:1000 in T-PBS). The images were captured with Leica DFS490 digital camera (Leica Microsystems, Wetzlar, Germany) at 100x magnification using Leica DMI 4000 fluorescent microscope (Leica Microsystems, Wetzlar, Germany).

### Quantitative Assessment of Cerebral β-Amyloidosis

The sections spanning brain from 0.48 to 1.92 mm relative to the midline in lateral stereotaxic coordinates were used to quantify congophilic amyloid plaques in the hippocampus. Every 15th section was analyzed, yielding 10 sections per animal. Amyloid plaques were identified by Congo Red staining and manually counted as described previously (Ninkina et al., 2009; Bachurin et al., 2012) using Zeiss Axiovert 200 M microscope with 10×, 20×, and 40× objectives (Carl Zeiss, Oberkochen, Germany), with examination under bright-field and between crossed polarizers. Amyloid plaques of all sizes were accepted for counting if they were visible and met the following requirements: red coloring under bright-field and the green birefringence in polarized light. Analyses were undertaken by two researchers independently (EB, SK). To determine the reproducibility of the plaques counts, an intra-class correlation (ICC) was calculated yielding good inter-rater reliability between the two researchers (ICC > 0.85). For each group of mice, the average values (± SD) of the plaques number per section were calculated.

### Hydrolytic Activity of Na^+^/K^+^-ATPase

The purified preparation of Na^+^/K^+^-ATPase (α1β1 isozyme) was obtained from duck salt glands as described elsewhere (Petrushanko et al., 2016). The purity grade of Na^+^/K^+^-ATPase was 99% and specific activity of the enzyme reached ∼2400 μmol of Pi (mg of protein × h)^−1^ at 37°C.

Hydrolytic activity of Na^+^/K^+^-ATPase in the purified preparation was measured as ouabain-sensitive (1 mM) ATP cleavage in the reaction medium containing 130 mM NaCl, 20 mM KCl, 3 mM MgCl_2_, 3 mM ATP, and 30 mM imidazole, pH 7.4, 37°C as elsewhere (Petrushanko et al., 2016). Stock solutions containing Aβ_42_ and pS8-Aβ_42_ peptides, prepared as described above, were added to the reaction medium (without ATP) up to a concentration of 40 μM. An equivalent amount of DMSO was added to the control samples. Following 3–60 min of pre-incubation with amyloid peptides, the enzymatic reaction was started by adding ATP.

### Modeling of the Structure of pS8-Aβ42:Na^+/^K^+^-ATPase Complex

Model of the pS8-Aβ_42_ peptide was constructed using as templates the Aβ_42_ structure modeled in Petrushanko et al. (2016). The resulting model of pS8-Aβ_42_ was minimized in the AMBER99 force field with the AutoDockTools program. Modeling of the pS8-Aβ_42_:Na^+^/K^+^-ATPase complex was performed using the structure of Na^+^/K^+^-ATPase from shark glands 2zxe (PDB id) solved at 2.4 Å resolution (Shinoda et al., 2009), and the modeled structure of pS8-Aβ_42_. Docking has been carried out with VinaAutoDock program (Trott and Olson, 2010), and the docking was constrained to cover only the extracellular part of the protein.

### Isothermal Titration Calorimetry (ITC)

The thermodynamic parameters of amyloid peptides binding to Na^+^/K^+^-ATPase were measured using a MicroCal iTC200 instrument, as described elsewhere (Mitkevich et al., 2012; Petrushanko et al., 2014). Experiments were carried out at 25°C in 10 mM imidazole buffer (pH 7.5), containing 130 mM NaCl, 30 mM KCl, 3 mM MgCl_2_. Aliquots (2.6 μl) of ligands were injected into a 0.2-ml cell containing protein solution to achieve a complete binding isotherm. Protein concentration in the cell ranged from 10 to 20 μM, and ligand concentration in the syringe ranged from 100 to 200 μM. The resulting titration curves were fitted using the MicroCal Origin software, assuming one set of binding sites. Affinity constants (Ka), enthalpy variations (ΔH) and stoichiometry of binding (N) were determined and the Gibbs energy (ΔG) and entropy variations (ΔS) were calculated from the equation: ΔG = −RTlnKa = ΔH-TΔS.

### Statistical Methods Used for Data Analysis

Data are presented as means of at least three independent experiments ± SD. The Mann–Whitney test was used for pairwise comparison between examined groups of mice; *P* < 0.05 was considered significant. The comparison of Na^+^/K^+^-ATPase data groups was performed using one-way ANOVA with *post hoc* testing (using paired samples Student’s *t*-test with Bonferroni correction); after a Bonferroni correction a *P*-value < 0.016 was considered as statistically significant. Statistical analysis was performed using STATISTICA 8.0 (StatSoft, Inc., Tulsa, OK, United States).

## Results

### pS8-Aβ_42_ Suppresses Zinc-Dependent Aggregation of Aβ_42_

**FIGURE 1:**
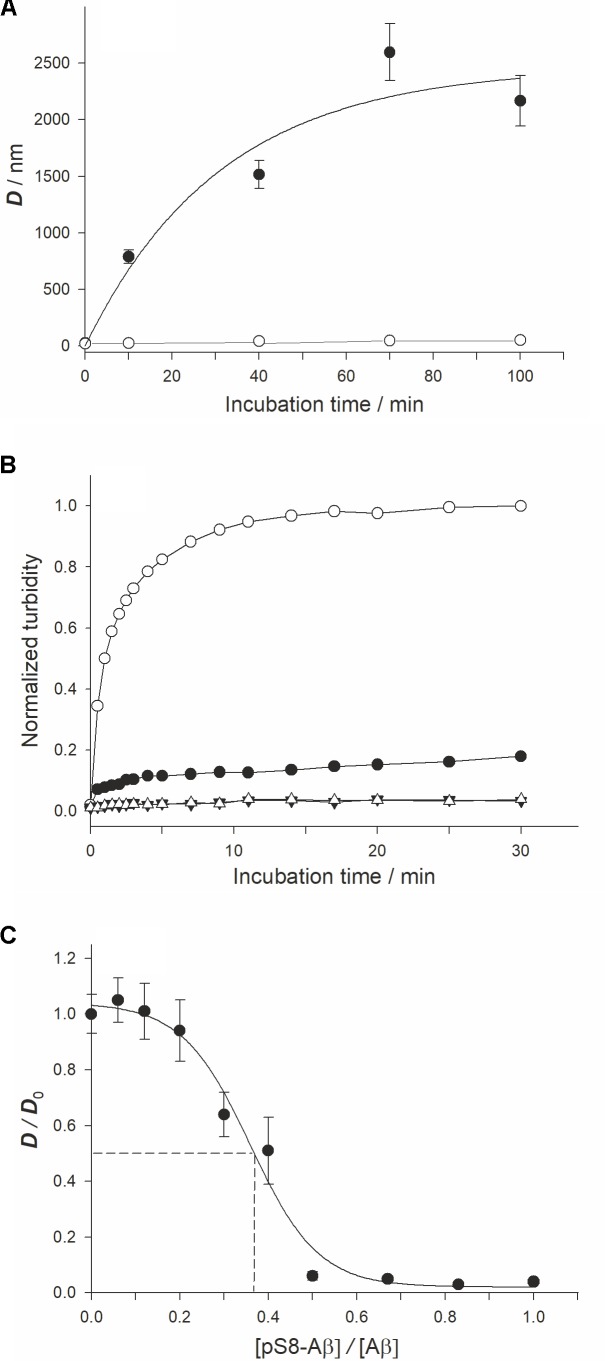
Characterization ofx the zinc-induced aggregation of Aβ_42_ and pS8-Aβ_42_ peptides. Measurements were performed in 10 mM HEPES (pH 7.3), 150 mM NaCl. **(A)** The characteristic diameter (*D*) of zinc-induced aggregates of Aβ_42_ and pS8-Aβ_42_ was monitored with DLS. White circles – Aβ_42_, black circles – pS8-Aβ_42_. Peptide and zinc ion concentrations – 25 and 50 μM, respectively. **(B)** Aβ solution turbidity (optical density at 405 nm, OD_405_) in the presence (circles) and absence (triangles) of zinc ions was monitored over 30 min. The normalized turbidity was obtained by dividing the OD_405_ values by the highest OD_405_ value observed (OD_405_ = 0.055). White symbols – Aβ_42_; Black symbols – pS8-Aβ_42_. Peptide and zinc concentrations as in **(A)**. **(C)** The relative size of zinc-induced Aβ aggregates at the incubation time of 70 min in solutions with different pS8-Aβ_42_/Aβ_42_ molar ratios. *D*_0_ – the size of zinc-induced aggregates in Aβ_42_ solution (without pS8-Aβ_42_) at this time point. Aβ_42_ and zinc ion concentrations were of 25 and 50 μM at each pS8-Aβ_42_/Aβ_42_ molar ratio tested. The dashed lines indicate IC_50_ (≈9 μM; this value corresponds to about one pS8-Aβ_42_ peptide per three Aβ_42_ peptides). Data are mean values for three independent experiments ± SD. Size of the white circles in **(A)** is larger than the error bars. Error bars for **(B)** are not shown.

Using DLS, we observed a time-dependent formation of Aβ_42_ or pS8-Aβ_42_ in the presence of Zn^2+^ (**Figure 1A**). Prior to zinc addition, only oligomers 20–30 nm in size were detected. In the absence of zinc ions, there were no appreciable changes in the characteristic size of both Aβ_42_ and pS8-Aβ_42_ oligomers during the 90 min incubation period (at 25°C and quiescent conditions). After 10 min of incubation with Zn^2+^, the characteristic diameter of Aβ_42_ aggregates reached 700–800 nm, and it became more than 2,000 nm by the end of the observation period (100 min). In contrast, pS8-Aβ_42_ did not form aggregates larger than 50 nm in the presence of zinc ions during the entire observation period. The results of the turbidity measurements support those of DLS experiments (**Figure 1B**): Ser8 phosphorylation substantially suppressed the propensity of Aβ peptides for zinc-triggered aggregation, which was manifested in the remarkably higher turbidity of the Aβ_42_/Zn^2+^ mixture than that of the pS8-Aβ_42_/Zn^2+^ mixture. The effect of Ser8 phosphorylation on zinc-induced Aβ aggregation was the opposite of that on spontaneous Aβ aggregation: as revealed in the ThT assay, in the absence of zinc ions, pS8-Aβ_42_ aggregates much faster than Aβ_42_ (**Supplementary Figure 1**). This result is consistent with those reported by Kumar et al. (2011, 2013).

To study a possible effect of pS8-Aβ_42_ on the zinc-induced aggregation of Aβ_42_, we determined the characteristic size of zinc-induced aggregates in a series of pS8-Aβ_42_/Aβ_42_ mixtures with different molar ratios of the peptides (**Figure 1C**) at the 70-min time point (the time point at which *D* reaches a plateau at a zinc/Aβ_42_ molar ratio of 1:3; **Supplementary Figure 2**). When equimolar mixtures of pS8-Aβ_42_ and Aβ_42_ (12.5 μM each) were tested in the presence of 50 μM Zn^2+^, the aggregate diameter decreased to (45 ± 10) nm. Thus, mixing of pS8-Aβ_42_ peptides with the unmodified peptide strongly suppressed the zinc-dependent aggregation of the latter. The half-maximal inhibitory concentration (IC_50_) for pS8-Aβ_42_ was estimated, under the conditions tested, to be approximately 9 μM (**Figure 1C**); this value corresponds to about one pS8-Aβ_42_ peptide per three Aβ_42_ peptides.

**FIGURE 2:**
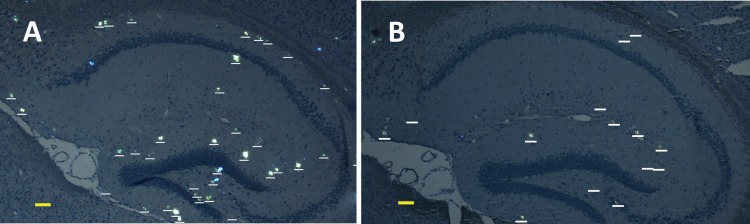
Representative polarized light micrographs of brain sections through the hippocampus for 8-months-old B6C3-Tg(APPswe, PSEN1dE9)85Dbo/j transgenic mice intravenously injected by sterile PS **(A)** or synthetic pS8-Aβ_42_ peptide **(B)**. Amyloid plaques are highlighted by white bars. Scale bars: **(A,B)**: 100 μm.

### Injection of pS8-Aβ_42_ Reduces the Amyloid Load in the Hippocampus of Transgenic AD Model Mice

We examined the ability of the synthetic pS8-Aβ_42_ peptide to reduce the cerebral amyloidogenesis in an APP/PS1 doubly transgenic murine model of AD. These mice have cognitive features of an AD-like pathology and accumulate significant amounts of dense-core congophilic amyloid plaques starting from 4 to 6 month of age, regardless of the sex (Borchelt et al., 1997; Garcia-Alloza et al., 2006). The experimental groups, which included male and female animals, were subjected to retro-orbital injections of peptide pS8-Aβ_42_ (10 μg in 125 μL of PS) starting from 2 months of age. After serial (at monthly intervals) inoculations with the peptide, the host mice were sacrificed at the age of 8 months. The brains were extracted, and sagittal brain sections (8-μm thick) were analyzed histochemically using Congo Red staining. The hippocampus was chosen as the target region for manual counting of the stained congophilic amyloid plaques using bright-field microscopy in the sections representing the brain layer located between 0.48 and 1.92 mm relative to the midline in lateral stereotaxic coordinates. The congophilic plaques found in the brains of all experimental animals were similar in terms of their location and size distribution in the brain parenchyma (**Figure 2**). Additionally, immunohistochemical characterization of the congophilic amyloid plaques revealed the presence of Aβ (**Supplementary Figure 3**). Quantitative analysis revealed a significantly lower number of congophilic amyloid plaques per section in the pS8-Aβ_42_-inoculated 8-month-old transgenic mice (−36.3%, *P* < 0.05) than that in the untreated littermates (**Figure 2** and **Table 1**).

**FIGURE 3:**
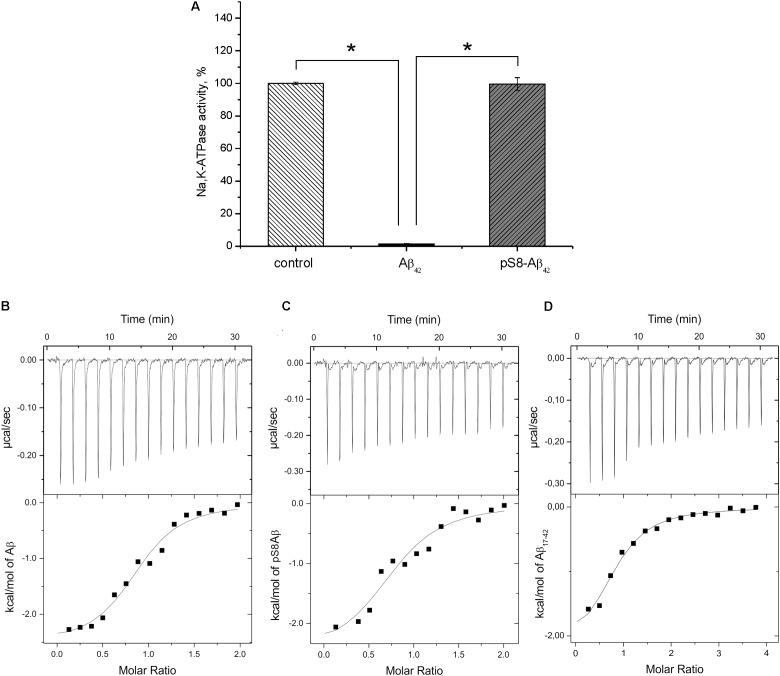
Interaction of Na^+^/K^+^-ATPase with beta-amyloid peptides. **(A)** Hydrolytic activity of purified Na^+/^K^+^-ATPase was measured after 60 min incubation with 40 μM of Aβ_42_ or pS8-Aβ_42_. The histogram represents the enzyme activity in the presence or absence of amyloid peptides, the enzyme activity without Aβ_42_ is accepted as 100%. Data are mean values for three independent experiments ± SD. Statistical analysis was performed using one-way ANOVA (*F* = 1894.8, degree of freedom 2, *P* < 0.00001) with *post hoc* testing (using paired samples Student’s *t*-test with Bonferroni correction); after a Bonferroni correction, a *P*-value < 0.016 was considered as statistically significant; ^∗^*P* < 0.001. ITC titration curves (upper panels) and binding isotherms (lower panels) for Aβ_42_ **(B)**, pS8-Aβ_42_ **(C)**, and Aβ_17-42_ **(D)** interaction with Na,K-ATPase at 25°C.

### pS8-Aβ_42_ Binds to Na^+^,K^+^-ATPase Without Inhibiting Its Hydrolytic Activity

For the measurements, we used purified Na^+^,K^+^-ATPase from duck salt glands, which is a homolog of the α1β1 human isozyme. Earlier, we have demonstrated that the unmodified Aβ_42_ inhibits the hydrolytic activity of the enzyme (Petrushanko et al., 2016). In contrast to Aβ_42_, which inhibited Na^+^,K^+^-ATPase after 60-min incubation with 40 μM peptide, pS8-Aβ_42_ had no effect on enzyme activity (**Figure 3A**). The interaction of Na^+^,K^+^-ATPase with the Aβ_42_ and pS8-Aβ_42_ peptides was measured by ITC (**Figures 3B,C**). The stoichiometry of binding to Na^+^,K^+^-ATPase was equal to 1 for both peptides, demonstrating that the peptides were predominantly in a monomeric state, as we have shown previously for Aβ_42_ (Petrushanko et al., 2016). The binding constants of Aβ_42_ and pS8-Aβ_42_ with the enzyme were close to each other, and the energy profiles [enthalpy (ΔH) and entropy (TΔS)] for binding of both peptides were practically the same (**Table 2**). This indicates that phosphorylation of Aβ does not affect the peptide interaction with Na^+^,K^+^-ATPase.

**Table 2:** Thermodynamic parameters of Aβ_42_ and pS8-Aβ_42_ binding to Na^+^/K^+^-ATPase determined by ITC at 25°C.

Ligand	Ka M^−1^	Kd μM	ΔH kcal/mol	TΔS kcal/mol	ΔG kcal/mol
Aβ_42_	7.7 × 10^5^	1.3	−2.54	5.48	−8.02
pS8-Aβ_42_	4.5 × 10^5^	2.2	−2.54	5.19	−7.73
Aβ_17–42_	2.6 × 10^5^	3.8	−2.27	5.13	−7.4
Aβ_16_	nd	–	nd	–	–

*Ka, affinity constant; standard deviation did not exceed 30%. Kd, dissociation constant; calculated as 1/K. ΔH, enthalpy variation; standard deviation did not exceed 10%. TΔS, entropy variation; calculated from the equation ΔG = ΔH-TΔS. ΔG, Gibbs energy; calculated from the equation ΔG = RTlnKa. nd, not detected.*

### Hydrophobic C-Terminal Domain of Aβ_42_ Is Responsible for Its Binding to Na^+^,K^+^-ATPase

**FIGURE 4:**
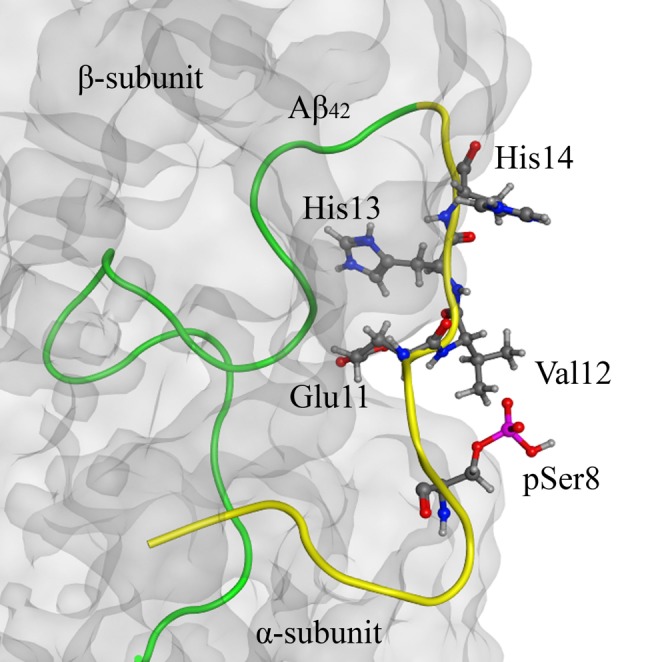
The model of Na^+^/K^+^-ATPase:pS8-Aβ_42_ peptide complex on the basis of 2zxe PDB structure. Na^+^/K^+^-ATPase is shown as a translucent gray molecular surface, C-terminal part (17–42) of pS8-Aβ_42_ peptide is shown in green and the N-terminal metal-binding domains (1–16) is shown in yellow. The phosphorylated Ser8 residue is located outside of the interaction region (highlighted in red). The zinc-binding site 11–14 and pSer8 residue are shown by the ball and stick representation.

Structure analysis of the Aβ_42_:Na^+^,K^+^-ATPase complex, which we performed earlier (Petrushanko et al., 2016), has shown that the Ser8 residue is located outside of the interaction site (**Figure 4**). Introduction of the phosphate group to Ser8 had no effect on the conformation of the Aβ_42_ polypeptide chain and did not change the interaction interface (**Figure 4**).

Binding parameters of the N-terminal zinc-binding domain, Aβ_1-16_, and C-terminal hydrophobic fragment, Aβ_17-42_, to Na^+^,K^+^-ATPase were determined by ITC. Binding was detectable for the 17–42 fragment (**Figure 3D**) but not for the 1–16 fragment (**Table 2**).

### Metal-Dependent Aggregation of Aβ_42_ and pS8-Aβ_42_ Correlates With Their Ability to Inhibit Na^+^,K^+^-ATPase

Using DLS, we showed that Mg^2+^ at concentrations of 3 mM and above induces the aggregation of Aβ_42_ after 10 min of incubation (**Supplementary Figure 4**). By contrast, no oligomers of pS8-Aβ_42_ were observed even after 20 min of incubation with 10 mM Mg^2+^. Since Na^+^,K^+^-ATPase activity was measured in a buffer containing 3 mM Mg^2+^, we hypothesized that Mg^2+^-dependent oligomers of Aβ_42_ are required to inhibit Na^+^,K^+^-ATPase and the absence of such aggregates in pS8-Aβ_42_ solution determines the absence of inhibition. To support this conclusion, we measured the Na^+^,K^+^-ATPase inhibition by Aβ_42_ at different time points and found that the degree of inhibition increases over time and plateaus after 30 min of incubation (**Supplementary Figure 5**). However, Aβ_42_ incubated in Mg^2+^-containing solution for 30 min (in the absence of Na^+^,K^+^-ATPase) did not inhibit Na^+^,K^+^-ATPase if the activity was measured immediately after the addition of the oligomers to the enzyme-containing solution (**Supplementary Figure 6**). Since both Aβ_42_ and pS8-Aβ_42_ are able to bind to Na^+^,K^+^-ATPase, we hypothesized that the initial binding of Aβ peptides to the enzyme acts as a seed for further aggregation of Aβ on the enzyme matrix and results in the inhibition of the enzyme. In the case of pS8-Aβ_42_, binding to the enzyme is not followed by oligomerization, which explains the absence of inhibition.

**FIGURE 5:**
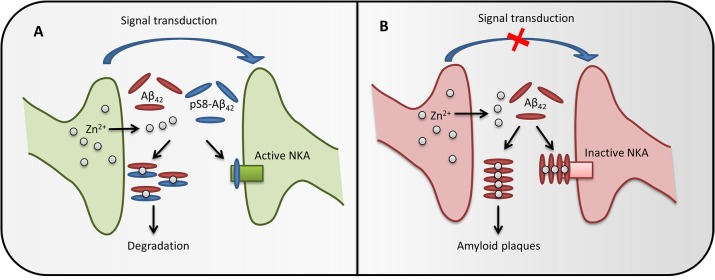
The possible role of pS8-Aβ_42_ in maintaining brain health. In healthy brain synapses **(A)**, the release of Zn^2+^ during synaptic transmission does not cause Aβ accumulation, as phosphorylated Aβ does not form zinc-induced aggregates and also prevents zinc-induced aggregation of the intact peptide through the formation of heterodimers. Phosphorylated Aβ does not inhibit Na^+^/K^+^-ATPase (NKA) and the heterodimers are easily cleared, therefore the synapse function is not impaired. If pS8-Aβ_42_ is depleted **(B)**, synaptic release of zinc ions promotes oligomerization of Aβ, inhibition of Na^+^,K^+^-ATPase and, eventually, leads to the amyloid plaques formation, which impairs the synapse function.

## Discussion

Since it was first proposed by Hardy in 1992, the amyloid hypothesis of AD has undergone a number of changes (Selkoe and Hardy, 2016). The focus of research has shifted from senile plaques to soluble toxic oligomers of Aβ, and the inherent role of the tau-protein in the AD pathogenesis has been elucidated. Accumulation and aggregation of Aβ are still considered triggers of the AD pathological cascade (Musiek and Holtzman, 2015); however, recent failures of monoclonal antibodies against Aβ in clinical trials call for reassessment of the role of Aβ in AD pathogenesis (Abbott and Dolgin, 2016). It is possible that the pivotal factor is not the total amount of Aβ in the blood but the range and relative quantities of modified Aβ species. A number of different modified Aβ forms, such as isomerized (Roher et al., 1993; Shimizu et al., 2000), pyroglutamylated (Wirths et al., 2009; Nussbaum et al., 2012), truncated (Kummer and Heneka, 2014), and other peptides, have been identified in senile plaques. Previously identified chemical modifications of Aβ seem to increase the pathogenic properties of the peptide. However, based on the present data, Ser8 phosphorylation could be the first identified modification that reverses some disease-associated properties of Aβ.

Protein phosphorylation is a ubiquitous modification, which tightly and precisely regulates the structural and functional characteristics of proteins (Hunter, 1995; Cohen, 2001). Aberrant protein phosphorylation is a disease-modifying factor, one of the most prominent examples of which is the hyperphosphorylation of tau in AD (Cohen, 2001; Ballatore et al., 2007). Recently, pS8-Aβ was obtained *in vitro* by Aβ phosphorylation with protein kinase A and was subsequently identified *in vivo* (Kumar et al., 2011, 2013). Based on the oligomeric state of pS8-Aβ derived from brain tissue of AD model mice (Kumar et al., 2013) and accelerated aggregation of pS8-Aβ_40_ *in vitro*, Kumar et al. (2011) have proposed that pS8-Aβ represents a potentially pathogenic agent in AD. However, the aggregation of pS8-Aβ was previously studied in the absence of divalent cations, particularly zinc ions, which are involved in both physiological processes and AD pathogenesis (Takeda, 2000; Frederickson et al., 2005; Kumar et al., 2011; Jamasbi et al., 2017). In a complex with Aβ, zinc ions can form seeds of pathogenic aggregation (Frederickson and Bush, 2001; Miller et al., 2010; Bush, 2012). This is especially likely to occur in synapses, where concentrations of Zn^2+^ may reach 100s of micromoles per liter (Paoletti et al., 2009).

We have previously shown that phosphorylation of Aβ at Ser8 leads to an increase in the zinc-dependent dimerization of Aβ (Kulikova et al., 2014). Zinc-induced dimerization of the unmodified Aβ occurs through residues 11EVHH14 (Kozin et al., 2011), and the His6 residue is recruited for further oligomerization (Istrate et al., 2016). However, in pS8-Aβ dimers, His6 forms an additional intramolecular Zn^2+^-binding site with the pSer8 residue, which thereby excludes the His6 residue from further oligomerization (Kulikova et al., 2014). Thus, phosphorylation should lead to a decrease in the ability of Aβ to form zinc-induced aggregates. We investigated the effect of Ser8 phosphorylation on the pathogenic properties of the Aβ species Aβ_42_, which is more prone to aggregation than Aβ_40_ and seems to trigger the disease in a number of models (Jarrett et al., 1993; Johnson-Wood et al., 1997; Gouras et al., 2000). As expected, Ser8 phosphorylation reduced the zinc-driven aggregation of Aβ_42_. The formation of aggregates in an equimolar mixture of pS8-Aβ_42_ and Aβ_42_ was also dramatically suppressed. This may be due to the fact that pS8-Aβ_42_ forms zinc-induced heterodimers with the unmodified Aβ_42_, which are not capable of further aggregation. Formation of heterodimers between the metal-binding domains of Aβ and pS8-Aβ was previously observed *in vitro* (Mezentsev et al., 2016).

We hypothesized that *in vitro* inhibition of zinc-dependent aggregation by pS8-Aβ_42_ will result in an anti-amyloidogenic effect *in vivo*. To test the hypothesis, we studied the effect of retro-orbital injections of pS8-Aβ_42_ on the progression of cerebral amyloidosis in a murine model of AD, B6C3-Tg(*APPswe,PSEN1dE9*)85Dbo/j. We have previously found that retro-orbital injection of isoD7-Aβ_42_, but not of Aβ_42_, promoted the amyloid plaque formation in mice of this line (Kozin et al., 2013). It was the first evidence of the facilitation of cerebral amyloidosis by a synthetic Aβ species injected into the bloodstream. The ability of blood-derived Aβ to induce AD-like pathology has recently been confirmed in a murine parabiosis model (Bu et al., 2017); however, the authors have not identified the Aβ species that triggered the pathogenesis. It is important to mention that isoD7-Aβ_42_ exhibits increased zinc-dependent oligomerization *in vitro* (Istrate et al., 2016), and may serve as a seed of zinc-dependent aggregation of Aβ in brains of transgenic mice. In the case of pS8-Aβ_42_, which is unable to form zinc-dependent aggregates, we expected to observe an opposite effect. Indeed, the number of plaques in the hippocampus of the transgenic mice that received pS8-Aβ_42_ injections was approximately two-third of that in the mice that received PS injections. Apparently, pS8-Aβ_42_ is unable to serve as an aggregation seed *in vivo* and also partially prevents the amyloidogenic aggregation of the endogenous Aβ peptides. Based on zinc-dependent aggregation of the Aβ_42_/pS8-Aβ_42_ mixture *in vitro*, it is likely that pS8-Aβ_42_ forms heterodimers with Aβ_42_ in the nerve tissue, which prevents the formation of senile plaques. This indicates that bloodstream-derived Aβ can serve not only as a trigger but also as an obstacle for cerebral amyloidosis progression, depending on the Aβ species composition. These findings further support the role of zinc-induced aggregation as an important event in the amyloidogenic process.

It is known that cognitive deficits in AD or in corresponding models do not always correlate with the appearance of amyloid plaques and often appear before Aβ aggregates can be detected (Musiek and Holtzman, 2015). Such effects are associated with soluble toxic oligomers of the Aβ peptide (Haass and Selkoe, 2007) or with receptor-mediated effects of Aβ (Dinamarca et al., 2012). Recently, we have shown that Aβ can bind to Na^+^,K^+^-ATPase (Petrushanko et al., 2016), whose activity is critically important for maintaining electrogenic properties of neurons. Here, we found that binding of monomeric Aβ to the enzyme and subsequent oligomerization of the peptide on the Aβ:Na^+^,K^+^-ATPase matrix leads to the inhibition of enzyme activity. This observation provides a possible explanation for the decrease in the activity of Na^+^,K^+^-ATPase in brain tissues of AD patients (Zhang et al., 2013; Kairane et al., 2014; Petrushanko et al., 2016). Unlike the unmodified peptide, pS8-Aβ_42_ does not show an inhibitory effect on Na^+^,K^+^-ATPase. Surprisingly, the binding parameters of pS8-Aβ_42_ to the enzyme were almost identical to those of the unmodified peptide; thus, it is not the initial binding that defines the inhibitory properties of Aβ_42_ toward Na^+^,K^+^-ATPase. We further showed that the N-terminal domain of Aβ_42_ (including the pS8 residue) is not involved in the initial binding and is probably exposed to the solution. Since the N-terminal domain of the peptide governs its metal-dependent oligomerization, we suggest that the inhibition of Na^+^,K^+^-ATPase is caused by the metal-dependent formation of Aβ oligomers seeded by the solution-exposed N-terminal domain of the first Na,K-ATPase-bound peptide. Phosphorylation of the peptide at Ser8 seems to interfere with this process, which may correspond to its inability to form oligomers triggered by Mg^2+^ (present in Na^+^,K^+^-ATPase activity measurements buffer) or by Zn^2+^. Thus, a decrease in the level of phosphorylation of the peptide in the elderly may lead to the inhibition of Na^+^,K^+^-ATPase, development of neurotoxic effects, and the disruption of nerve transduction long before the appearance of amyloid aggregates. The possible role of Aβ phosphorylation in the brain is presented at **Figure 5**.

## Conclusion

In this study, we demonstrated that the phosphorylation of Aβ_42_ at Ser8 changes its properties of zinc-driven aggregation, inhibition of Na^+^/K^+^-ATPase, and amyloidogenicity. The obtained data indicate that the phosphorylation of Aβ_42_ neutralizes some of its AD-associated properties. Our findings provide the basis for discussion about the role of Ser8 phosphorylation in Aβ_,_ which was previously considered only as a pathogenic modification. The anti-amyloidogenic properties of pS8-Aβ_42_ *in vivo* support the intrinsic role of zinc-mediated aggregation in the formation of the senile plaques. Further studies addressing the role of pS8-Aβ in the human brain are needed for better clarification of both the significance of pS8-Aβ and the relevance of the obtained data to AD.

## Author Contributions

EB and IP performed most of the experiments with contributions from GT, AC, and SR. EB, IP, and VM drafted the paper. SK, OL, and AM contributed the conception and design of the study. All authors contributed to manuscript revision, read and approved the submitted version.

## Conflict of Interest Statement

The authors declare that the research was conducted in the absence of any commercial or financial relationships that could be construed as a potential conflict of interest.

## Abbreviations

Aβ: β-amyloid peptide
Aβ_42_: Aβ_16_, Aβ_17-42_, Aβ_40,_ β-amyloid peptide isoforms 1–42, 1–16, 17–42, 1–40, respectively
AD: Alzheimer’s disease
DLS: dynamic light scattering
isoD7-Aβ: isomerized Asp7-containing β-amyloid peptide
ITC: isothermal titration calorimetry
PDB: Protein Data Bank
PS: physiological saline
pS8-Aβ: phosphorylated Ser8-containing β-amyloid peptide
sAD: sporadic Alzheimer’s disease.
