# Supplementary material for: Phosphorylation of the Amyloid-Beta Peptide Inhibits Zinc-Dependent Aggregation, Prevents Na,K-ATPase Inhibition, and Reduces Cerebral Plaque Deposition
Source: Front Mol Neurosci. 2018 Aug 29;11:302. doi: 10.3389/fnmol.2018.00302 (PMC6123382; doi:10.3389/fnmol.2018.00302)
Supplement: Supplementary file 1 [file Data_Sheet_1.pdf]

Supplementary Material

Phosphorylation of the amyloid-beta peptide inhibits zinc-dependent aggregation, prevents Na,K-ATPase inhibition, and reduces cerebral plaque deposition

Evgeny P. Barykin, Irina Y. Petrushanko, Sergey A. Kozin, Georgy B. Telegin, Alexander S. Chernov, Olga D. Lopina, Sergey P. Radko, Vladimir A. Mitkevich, Alexander A. Makarov*
*** Correspondence:** Prof. Alexander Makarov, aamakarov@eimb.ru

Supplementary Figures

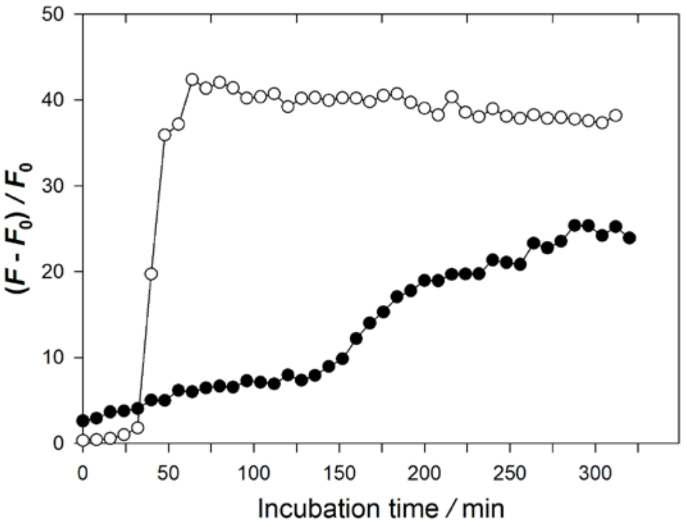

Supplementary Figure 1. Thioflavin T fluorescence (F-F$_0$)/F$_0$ in solutions of Aβ_{42} peptide (black symbols) and pS8-Aβ_{42} peptide (white symbols) was monitored over 300 min. Fluorescence measurements were carried out on the Infinite M200 PRO microplate reader (TECAN, Switzerland) using Corning 96-well microplates. The excitation and emission wavelengths were set at 450 and 482 nm, respectively. To test the self-aggregation of Aβ peptide isoforms, 100-μL aliquots of Aβ solutions (peptide concentration – 30 μM) were mixed in wells with 20 μL of the thioflavin T (ThT) solution in buffer H (ThT concentration – 150 μM), followed by the incubation at 37°C with constant agitation. The fluorescence measurements were started immediately after preparation of Aβ/ThT mixtures. Values of fluorescence in wells containing buffer alone were subtracted from those in wells containing ThT. The relative changes of ThT fluorescence intensity were calculated as (F - F0) / F0, where F and F0 are fluorescence intensities of ThT in the presence and absence of Aβ peptides, respectively. The mean values of triplicate measurements are shown.

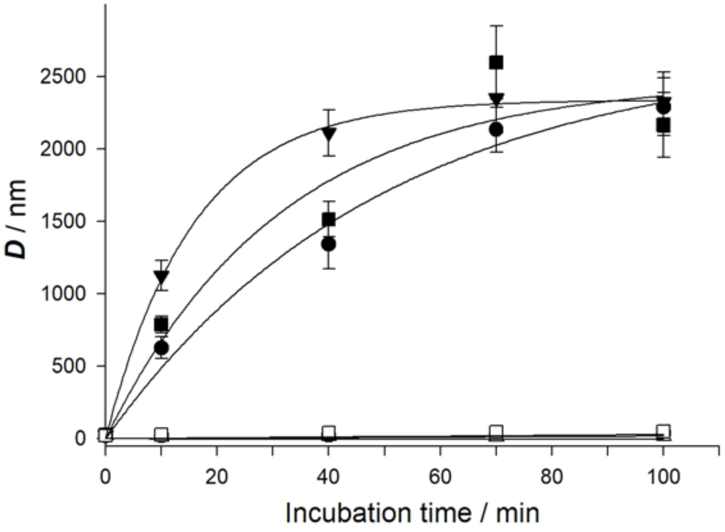

Supplementary Figure 2. Characteristic diameter (*D*) of zinc-induced aggregates of Aβ_{42} and pS8-Aβ_{42} peptides was monitored with DLS over 100 min. black symbols – Aβ_{42}, white symbols – pS8-Aβ_{42}. Circles, squares, and triangles – zinc/peptide molar ratios of 1, 2, and 3, respectively. Peptide concentration – 25 µM. Buffer – 10 mM HEPES (pH 7.4), 50 mM NaCl. Data are mean values for three independent experiments ± SD. Size of some symbols is larger than the error bars.

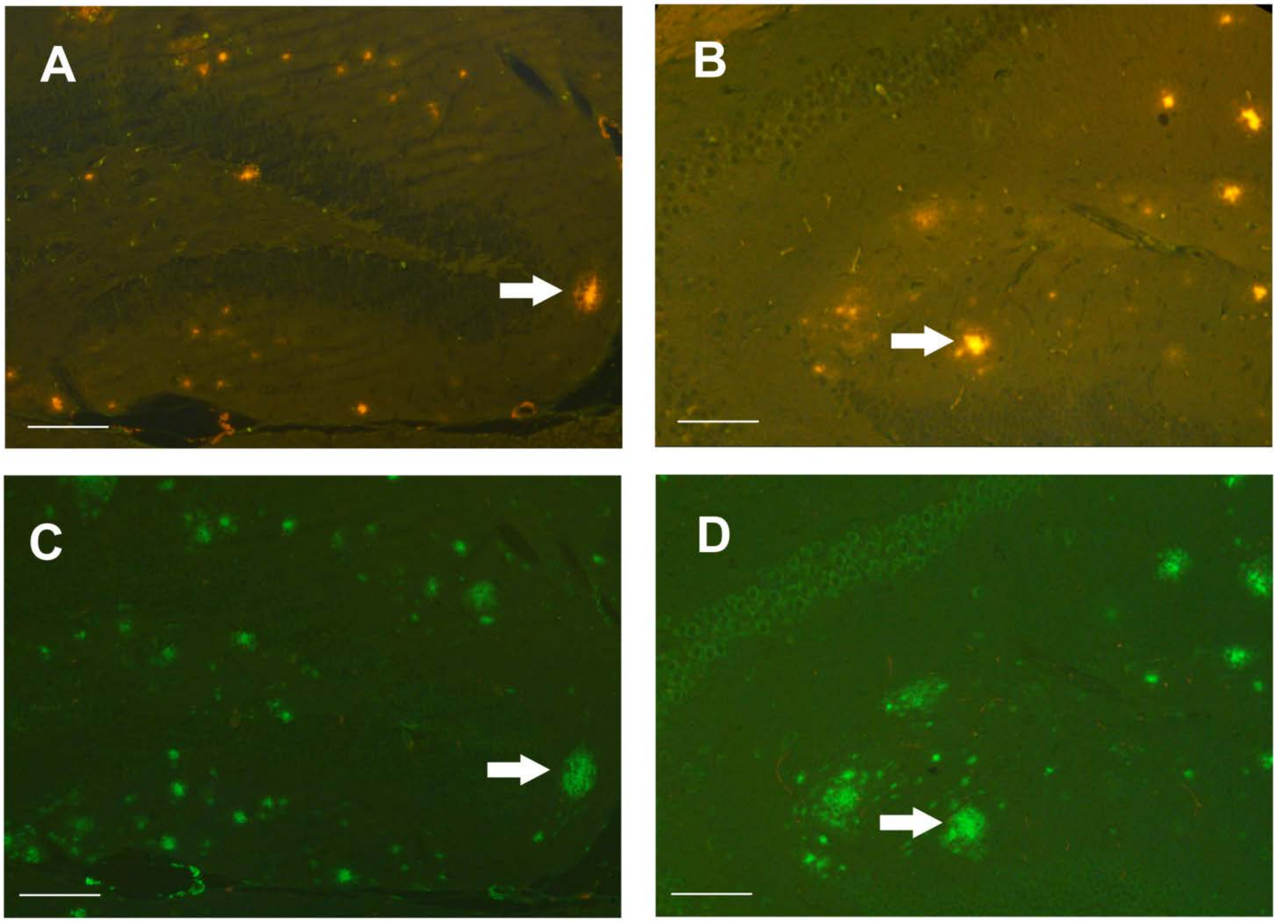

Supplementary Figure 3. Representative fluorescent micrographs of brain sections through the hippocampus for 8-month-old B6C3-Tg(APPswe,PSEN1dE9)85Dbo/j transgenic mice intravenously injected with sterile physiological saline (**A, C**) or synthetic pS8-Aβ_{42} peptide (**B, D**). Counterstaining of adjacent sections of the brain in the dentate gyrus of the hippocampus by Congo Red dye (**A, B**), and immunohistochemical staining by specific antibodies to Aβ (**C, D**). Selected amyloid inclusions are indicated by white arrows. Scale bars: (**A, B, C, D**) 100 μm.

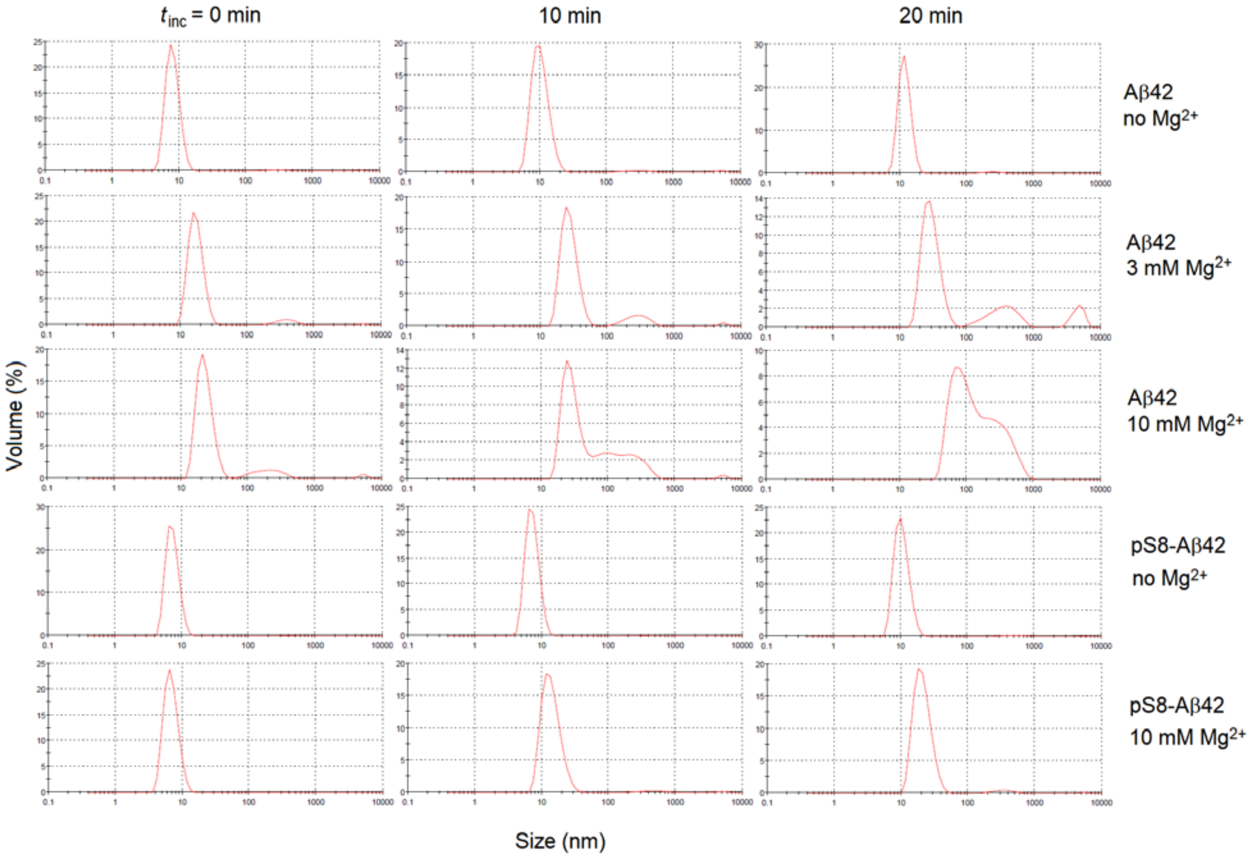

Supplementary Figure 4. The graphic output of the Zetasizer Nano ZS apparatus: Representative size distributions by volume (fraction of total particle volume, %, occupied by particles of a given diameter) for $A\beta_{42}$ and pS8-$A\beta_{42}$ isoforms in the absence and the presence of magnesium ions at various incubation times. Peptide concentration – 25 µM. Buffer – 10 mM HEPES (pH 7.4), 150 mM NaCl. DLS measurements were started immediately after the addition of magnesium ions. Peptide solutions were incubated at 25°C under quiescent conditions.

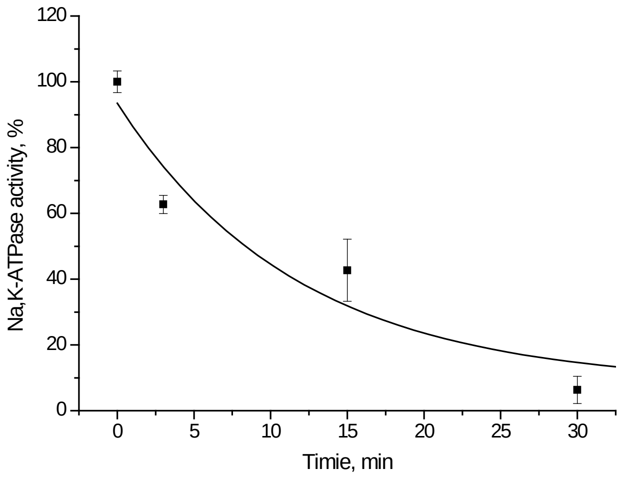

Supplementary Figure 5. Effect of Aβ42 on hydrolytic activity of Na,K-ATPase in solution. Hydrolytic activity of Na,K-ATPase after 0, 3, 15, 30 min incubation with 30 µM Aβ42. Enzyme activity without Aβ42 is accepted as 100%. Data are mean values for three independent experiments ± SD. Curve was fitted by one phase exponential decay equation using Origin program.

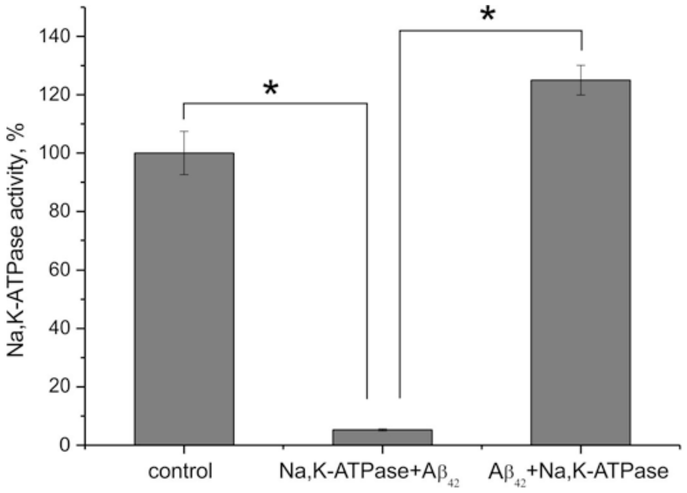

Supplementary Figure 6. Effect of Aβ42 on hydrolytic activity of Na,K-ATPase in solution. The hydrolytic activity of Na^+,K^+-ATPase was measured after 30 min incubation with 40 µM of Aβ42 (Na,K-ATPase + Aβ42). Alternatively, 30 µM Aβ42 was incubated in a solution for 30 min, after which Na^+,K^+-ATPase was added and its activity was measured immediately (Aβ42+Na,K-ATPase). Enzyme activity without Aβ42 is accepted as 100%. Data are mean values for three independent experiments ± SD. Statistical analysis was performed using one-way ANOVA (F=414.4, degree of freedom 2, P<0.00001) with post-hoc testing (using paired samples Student's t-test with Bonferroni correction); after a Bonferroni correction, a P value <0.016 was considered as statistically significant; * P< 0.001.
